# Transfection of Peripheral Blood Monocytes with *SOX2* Enhances Multipotency, Proliferation, and Redifferentiation into Neohepatocytes and Insulin-Producing Cells

**DOI:** 10.1155/2018/4271875

**Published:** 2018-10-04

**Authors:** Ayman Hyder, Sabrina Ehnert, Fred Fändrich, Hendrik Ungefroren

**Affiliations:** ^1^Faculty of Science, Damietta University, Damietta 34517, Egypt; ^2^Siegfried Weller Institute for Trauma Research, Eberhard Karls University, Tübingen, Germany; ^3^Institute for Applied Cell Therapy, University Hospital Schleswig-Holstein, Campus Kiel, Kiel, Germany; ^4^First Department of Medicine, University Clinic Schleswig Holstein, Lübeck, Germany

## Abstract

Following a several-day incubation in medium containing IL-3 and M-CSF to generate a more plastic intermediate “reprogrammed multipotent cells of monocytic origin (RMCMO),” peripheral blood mononuclear cells (PBMCs) can be efficiently converted to hepatocyte-like cells (neohepatocytes) and insulin-producing cells. However, continuous efforts are devoted to enhancing the proliferative capacity of these multipotent cells while maintaining or further increasing their redifferentiation potential. In the present work, PBMCs were transfected with one pluripotency gene (*SOX2*) and the resulting RMCMO compared to standard RMCMO with respect to cell viability, proliferative activity, and redifferentiation potential. Ectopic *SOX2* expression increased the number of viable RMCMO, activated cell cycle genes, and enhanced proliferation as shown by quantitative RT-PCR and Ki67 immunofluorescent staining, respectively. Redifferentiation of RMCMO derived from *SOX2*-transfected PBMCs to neohepatocytes was more complete in comparison to control cells as revealed by higher urea and glucose secretion, increased activity of cytochrome P450 isoforms, and a phase II enzyme, while the same was true for insulin-producing cells as assessed by the expression of *INS*, *PDX1*, and *GLUT2* and glucose-stimulated insulin secretion. Our results indicate that *SOX2* transfection increases both multipotency and proliferation of RMCMO, eventually allowing production of neohepatocytes and insulin-producing cells of higher quality and quantity for transplantation purposes.

## 1. Introduction

Several studies have shown that hepatocyte-like cells can be generated from peripheral blood mononuclear cells (PBMCs) [1–4]. The procedure described by Ruhnke and colleagues initially involved a *de*differentiation step of the PBMCs in vitro to produce a cell type with stem cell-like characteristics termed “reprogrammed multipotent cells of monocytic origin” (RMCMO, formerly PCMO). This step was followed by *re*differentiation of RMCMO to either hepatocyte-like cells (neohepatocytes [3, 4]) or insulin-producing cells [3] with appropriate differentiation media.

The generation of neohepatocytes or insulin-producing cells may have impact on treatment of end-stage liver diseases or diabetes. Since transplantation of liver tissue or pancreatic islets is hampered by the shortage of donor tissues [5] and the lack of methodology to maintain primary hepatocytes and pancreatic *β* cells in a differentiated state in long-term in vitro culture [6], PBMCs may represent, after their tissue-specific *re*differentiation, a virtually unlimited source of cells for autologous transplantation. However, after the two-step reprogramming protocol of these cells, it was found that cell yields of neohepatocytes and insulin-producing cells were insufficient for transplantation purposes. A series of studies has been carried out with the goal of increasing the proliferative potential of these cells, including treatment with epidermal growth factor (EGF) during the redifferentiation step [6], or activin (s) or TGF-*β* during the *de*differentiation step [7]. However, these efforts resulted in only limited success.

One possibility to optimize neohepatocyte cell numbers is by increasing the plasticity of the RMCMO. Enhancing their stem cell features may induce their capacity for self-renewal until *re*differentiation is initiated. Application of the induced pluripotent stem cell (iPS) technology [8], in which embryonic stem cell-like cells have been generated from differentiated adult cells after introduction of four specific pluripotency genes, may enhance the plasticity of RMCMO leading to more efficient neohepatocyte redifferentiation. This technique has become safer and simpler by transferring the four genes by means of a plasmid vector rather than a virus [9, 10].

In the present study, the aim was to apply the iPS technology and generate iPS cells from PBMCs to increase proliferation and plasticity of the resulting RMCMO. However, transfecting the same plasmid used by Yu and colleagues [10] was unsuccessful due to a dramatic decrease in cell viability. Nevertheless, we were able to establish a transfection protocol with a plasmid encoding only *SOX2* that resulted in increased RMCMO proliferation and redifferentiation potential.

## 2. Materials and Methods

### 2.1. PBMC Isolation and Generation of RMCMO

PBMCs were isolated on day 0 from buffy coats of healthy donors by Histopaque density gradient centrifugation and further purified by adherence to T-75 culture flasks (Cell+, Sarstedt, Numbrecht, Germany) for 1–2 h in RPMI 1640 medium containing 10% human serum (Lonza, Cologne, Germany), 2 mmol/l glutamine, 100 U/ml penicillin, and 100 *μ*g/ml streptomycin (Invitrogen, Darmstadt, Germany). Nonadherent cells were removed after 2 h, leaving the adherent PBMC fraction which consists of 60–70% CD14+ cells [3]. Subsequently, RMCMO were generated according to a previously published protocol [3] by culturing the adherent monocytes for 4 days in RPMI 1640 medium supplemented with 5 ng/ml M-CSF and 0.4 ng/ml IL-3 (both from R&D Systems, Wiesbaden, Germany), 90 *μ*M 2-mercaptoethanol, and 10% human AB serum (Lonza, Verbier, Belgium).

### 2.2. *SOX2* Cloning

*Homo sapiens* SRY- (sex determining region Y-) box 2 (*SOX2*, NM_003106.2) complete sequence was isolated from the plasmid pEP4 E02S EN2K provided by Addgene (Cambridge, MA, USA). The following primers were designed to amplify *SOX2* sequence: CTG*GAATTC*ATGTACAACATGATGGAG, a forward primer with an *Eco*R1 restriction site and CAG*CTCGAG*TCACATGTGTGAGAGGG, a reverse primer with an *Xho*1 restriction site. The same restriction enzymes were used to cut the recipient plasmid pCAGGS [11]. PCR was performed using high-fidelity PCR mix, and both the product and vector were digested using FastDigest and the restriction enzymes *Eco*R1 and *Xho*1 (all from Fermentas/Thermo Fisher Scientific, Karlsruhe, Germany). Both the insert and vector were ligated using T4-Ligase Rapid kit (Fermentas). Ligation product was transformed overnight in DH5a *E. coli*, and the *SOX2* clones were then isolated using Fermentas jet plasmid miniprep. The identity of the product was verified by sequencing (MWG Biotech, Ebersberg, Germany).

### 2.3. Transfection

The plasmid pEP4 E02S EN2K was provided by Addgene and was originally deposited by Prof. James Thomson's lab [10]. It is used in the derivation of human iPS cells and expresses 4 pluripotency transcription factors: OCT3/4, SOX2, NANOG, and KLF4. The plasmid pCAGGS-sox2 was cloned as described. Lipofectamine 2000 (Invitrogen/Thermo Fisher Scientific, Germany) was used to transfect cultured PBMCs in 6-well plates on day 1 of culture according to manufacturers' instructions. Control cells were transfected with empty plasmids. Cell viability and counts were assessed two days later (on day 3) by Trypan blue staining, while parallel samples were subjected to RNA isolation and quantitative real-time RT-PCR (qPCR) to assess *SOX2* expression. Both control and *SOX2*-transfected cells were then cultured for another 4 days (days 3–7) in dedifferentiation medium to generate RMCMO as described before [6].

### 2.4. RNA Extraction and qPCR

RNA isolation was performed using the GeneJet purification kit (Fermentas). 1 *μ*g of total RNA was reverse transcribed to cDNA using the high-capacity reverse transcription kit (Applied Biosystems/Thermo Fisher Scientific, Germany). Gene expression was quantified by qPCR using SYBR Green (Applied Biosystems). Relative quantification was performed by ∆∆Ct method. To normalize gene expression data, amplification of the housekeeping gene *β*-actin was used as an internal control. Primers used for PCR amplification were *ACTB* forward GATATCGCTGCGCTCGTC, reverse TCCATATCGTCCCAGTTGG; *SOX2* forward TGATGGAGACGGAGCTGAAG, reverse GCTTGCTGATCTCCGAGTTG; *NANOG* forward GATTTGTGGGCCTGAAGAAAACT, reverse AGGAGAGACAGTCTCCGTGTGAG; *ANAPC2* forward CCAGTACAGGCGGTGATCTT, reverse GCTCTCGTCGTCACTGTCAA; *ABL-1* forward AACACCCTAACCTGGTGCAG, reverse CAAGTGGTTCTCCCCTACCA; *CDC2* forward GGGGTCAGCTCGTTACTCAA, reverse GATGCTAGGCTTCCTGGTTTC; *CDK4* forward CTGACCGGGAGATCAAGGTA, reverse AGCCAGCTTGACTGTTCCAC; *CDK6* forward TCCCAGGAGAAGAAGACTGG, reverse GGTCCTGGAAGTATGGGTGA; *INS* forward GGGGAACGAGGCTTCTTCTA, reverse AGTTGCAGTAGTTCTCCAGC; *PDX1* forward AAGTCTACCAAAGCTCACGC, reverse GTTCAACATGACAGCCAGCT; and *GLUT2* forward TTGGGCTGAGGAAGAGACTG, reverse AACCCCATCAAGAGAGCTCC.

### 2.5. Immunofluorescence

On day 7 of culture, adherent cells were fixed in 1% paraformaldehyde, incubated with anti-human CD14 antibody (BD Biosciences, Heidelberg, Germany) at room temperature for 2 h and Alexafluor 488-labeled secondary antibody (Invitrogen) for 1 h. After washing, cells were permeabilised using 0.5% Triton X-100 and incubated overnight with the anti-human Ki67 (BD Biosciences) at 4°C followed by Alexafluor 555-labeled secondary antibody (Invitrogen). Nuclei were stained with DAPI. Ki67-positive cells were counted and related to the cell count of CD14-positive PBMCs.

### 2.6. Redifferentiation of Transfected Cells to Neohepatocytes and Insulin-Producing Cells

Following completion of the dedifferentiation process of PBMCs on day 5 of culture, the resulting RMCMO were cultured for 2 weeks with either hepatocyte conditioning medium containing 3 ng/ml fibroblast growth factor-4 (FGF-4, R&D Systems, Wiesbaden, Germany) and 10% FBS for redifferentiation into neohepatocytes or islet cell-conditioning medium containing 10 ng/ml epidermal growth factor (EGF) and 20 ng/ml hepatocyte growth factor (HGF, both from Calbiochem, Darmstadt, Germany), 10 mmol/l nicotinamide (Sigma, Deisenhofen, Germany), and 5 mmol/l glucose for redifferentiation into insulin-producing cells [3]. The medium was changed every 3rd day. Redifferentiated cells were then subjected to analysis of hepatocyte or islet cell functions.

### 2.7. Functional Analyses of Neohepatocytes and Insulin-Producing Cells

The methodology for hepatocellular function was described in detail in our previous work [6]. For the measurement of insulin secretion, cells were washed twice with PBS and placed in 5% BSA blocking medium for 3 h, then incubated in secretion buffer containing different glucose concentrations for 2 h. The concentration of insulin in the medium was determined using ELISA kit (DRG diagnostics, Marburg, Germany) following the manufacturers' protocol. The method of rat islet isolation, culture, and rat insulin determination was described elsewhere [5]. Insulin-producing cells redifferentiated from RMCMO were also subjected to RNA extraction and conventional endpoint PCR to detect expression of *INS* (product size 202 bp), *PDX1* (product size 186 bp), and *GLUT2* (product size 192 bp) using the primers specified above.

### 2.8. Statistical Analysis

All samples were measured in duplicate. Values were expressed as mean ± SEM with *N* = 3 in all experiments. Statistical comparison between two groups was performed by Student's *t*-test. Statistical difference was considered significant if *p* < 0.05.

## 3. Results

### 3.1. Transfection with iPS Plasmid, but Not with a Plasmid Encoding *SOX2* Alone, Decreased PBMC Viability

**Figure 1:**
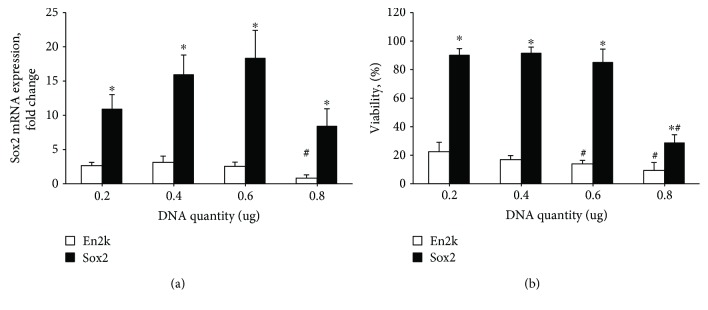
Transfection with *SOX2* plasmid increased *SOX2* mRNA expression and did not affect PBMC viability. PBMCs were transfected one day after plating with increasing concentrations of either the plasmid pEP4 E02S EN2K carrying the pluripotency genes *SOX2*, *KLF4*, *NANOG*, and *OCT3/4* (En2k) or pCAGGS plasmid carrying only *SOX2* (Sox2). The panels show *SOX2* mRNA expression as determined by qPCR (a) and the viability as determined by Trypan blue in the transfected cells by either plasmids (b) two days post transfection. Data are presented as mean ± SEM of *N* = 3. The symbol ^∗^ denotes a significantly higher Sox2 group value than En2k value (*t*-test). The symbol # indicates a significant difference to the respective cells which received 0.2 *μ*g DNA (Student's *t*-test after ANOVA *p* < 0.05).

Initially, it was planned to generate iPS cells from PBMCs. To accomplish this, we transfected PBMCs with the plasmid pEP4 E02S EN2K encoding *OCT3/4*, *NANOG*, *SOX2*, and *KLF4*. It was observed that cultured PBMCs could not withstand the transfection process, with their viability decreasing dramatically with most plasmid concentrations (Figure 1(b)). Moreover, the transfection was not efficient enough, as revealed by qPCR, and largely failed to increase the expression of pluripotency genes exemplified by the expression of *Sox2* (Figure 1(a)). This brought us to the conclusion that the surviving cells were those that have not taken up the plasmid DNA. Therefore, it was thought to decrease the toxicity of the transfection by decreasing the plasmid size. However, similar results were obtained after transfecting PBMCs with a plasmid containing only 3 genes (*OCT3/4*, *NANOG*, and *KLF4*) (data not shown), indicating that reducing plasmid size was unable to reduce toxicity.

Next, we constructed pCAGGS-sox2, a plasmid containing only the *SOX2* cDNA. Following transfection of PBMCs with increasing amounts of this plasmid, no significant alterations in their viability were observed until an amount of 0.6 *μ*g/well (Figure 1(b)). Meanwhile, the *SOX2* expression increased gradually and peaked at the same amount (Figure 1(a)), indicating a good efficiency of the *SOX2* transfection process.

### 3.2. *SOX2* Overexpression Induces RMCMO Proliferation

**Figure 2:**
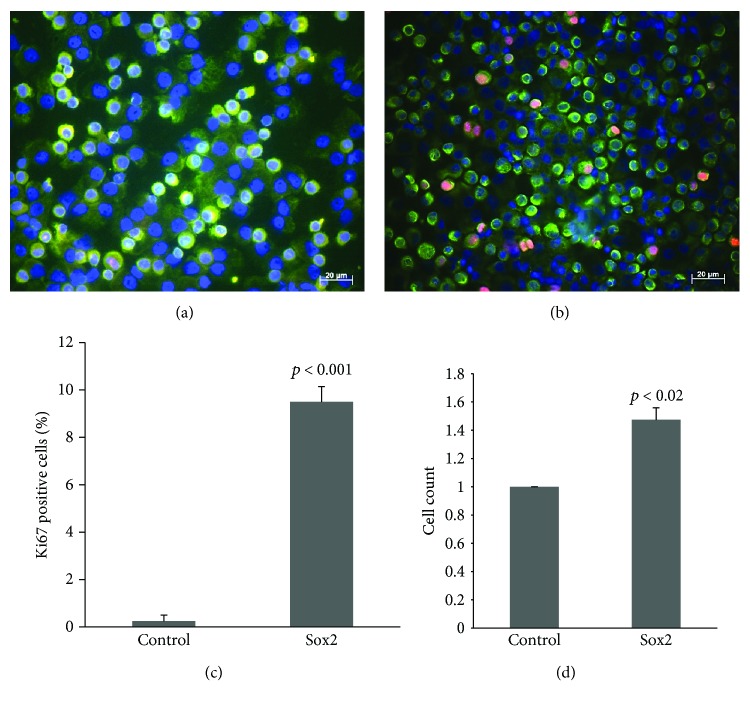
*SOX2* overexpression induces proliferation in RMCMO. Immunofluorescence of Ki67 expression (red) in normal RMCMO (a) or RMCMO generated after transfection of PBMCs with *SOX2* (b). Cells were also immunostained with anti-CD14 (green) as a monocyte-specific marker. Nuclei were stained with DAPI (blue). (c) Quantification of Ki67 in *SOX2*-overexpressing RMCMO. (d) RMCMO cell count (relative to control value set arbitrarily at 1) 4 days after *SOX2*-transfection. Control = empty vector-transfected RMCMO. Data (*N* = 3) are expressed as mean ± SEM. Statistical analysis was performed by unpaired Student's *t*-test.

Next, we examined the proliferative response of the PBMCs to *SOX2*-transfection. For this purpose, cells were stained by immunofluorescence for Ki67, a nuclear proliferation marker that is present only in mitotically active cells [12]. To assure the monocytic nature of the Ki67-positive cells, cultures were counterstained with anti-CD14, a cell surface marker of monocytes. The results showed no signs of proliferation in control RMCMOs (Figures 2(a) and 2(c)), while 8.5 ± 1% of *SOX2*-transfected RMCMO were positive for Ki67 (Figures 2(b) and 2(c)). Four days post transfection, cell counts were compared in both groups. It was found that cell numbers of *SOX2*-transfected cells significantly increased by 47.5 ± 9% over the control group (Figure 2(d)). Taken together, the data clearly show that *SOX2* can induce mitotic activity in RMCMO.

### 3.3. Ectopic *SOX2* Expression Is Associated with Transcriptional Upregulation of Genes Involved in Cell Cycle Regulation and Pluripotency

**Figure 3:**
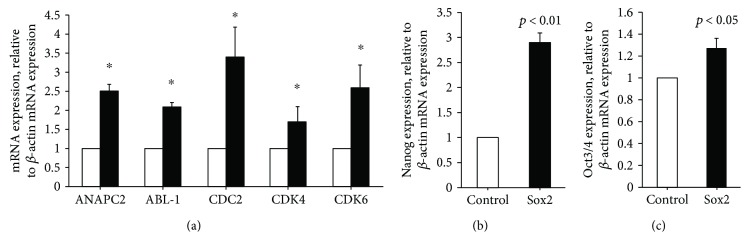
*SOX2* overexpression upregulates cell cycle and pluripotency gene expression in RMCMO. qPCR-based measurement of gene expression of five cell cycle regulatory genes (a) and the pluripotency genes, *NANOG* (b) and *OCT3/4* (c), in RMCMO derived from *SOX2*- (black columns) and control-transfected (white columns) PBMCs. Data for the gene of interest (mean ± SEM, *N* = 3, duplicates) were normalized to the gene expression level of *β*-actin. Statistical analysis between *SOX2*-transfected and empty vector-transfected cells was performed by unpaired Student's *t*-test.

The Ki67 data suggest that the increase in cell numbers in *SOX2*-overexpressing cells was the result of increased mitotic activity. Since mitotic activity involves transcriptional changes in key cell cycle regulators, we examined by qPCR analysis whether *SOX2*-induced proliferation was associated with the upregulation of genes that have regulatory roles in different stages of the cell cycle. As seen in Figure 3(a), *SOX2* overexpression significantly upregulated the mRNA expression of the ANAPC2, ABL-1, CDC2, CDK4, and CDK6 genes. These transcriptional effects on cell cycle genes are in agreement with the promitotic role of SOX2 and are likely mediated by its transcription factor function.

To reveal whether ectopic expression of *SOX2* in PBMCs impacts transcriptional regulation of other pluripotency-determining genes, we measured expression of *NANOG* and *OCT3/4* in the resulting RMCMO by qPCR. Interestingly, in *SOX2*-transfected cells, both *NANOG* and *OCT3/4* mRNA were significantly upregulated compared to control-transfectants (Figures 3(b) and 3(c)). This shows that *SOX2* does not only increase mitotic activity but also induces expression of other essential pluripotency-associated factors and, thereby, might enhance the plasticity/redifferentiation potential of RMCMO.

### 3.4. *SOX2* Enhances Hepatocyte-Specific Functions of Neohepatocytes

**Figure 4:**
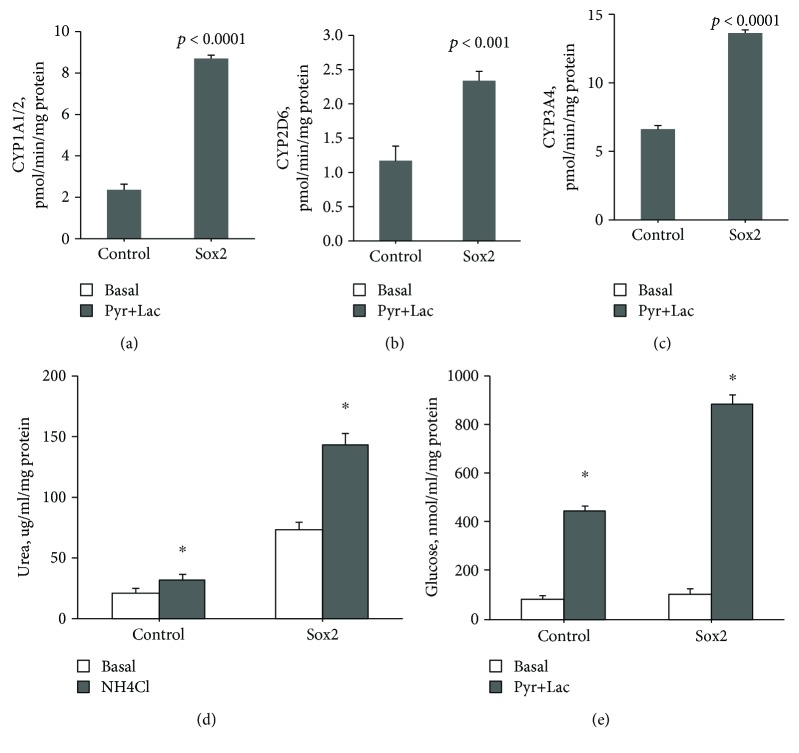
*SOX2*-overexpression improves hepatic cell function in RMCMO-derived neohepatocytes. RMCMO derived from *SOX2*-transfected PBMCs were cultured in hepatocyte conditioning medium for 2 weeks before subjecting them to analysis of cytochrome P450 (CYP) isoforms 1A1/2, 2D6, and 3A4, urea metabolism, and glucose metabolism. Activities and secretion levels were normalized to protein content (mg) of each sample. Data are presented as mean ± SEM of *N* = 3. Statistical analysis between *SOX2*-transfected and nontransfected control cells was performed by unpaired Student's *t*-test. Pyr, pyruvate; Lac, lactate.

The observation of *SOX2*-mediated upregulation of *OCT3/4* and *NANOG* suggested the possibility that *SOX2* can establish a regulatory network with other pluripotency factors that ultimately result in an increase in RMCMO plasticity. Hence, it was tested whether the overexpression of *SOX2* in PBMCs would change the functional parameters of RMCMO-derived neohepatocytes. After the 4-day culture period necessary for PBMC *de*differentiation to RMCMO, control and *SOX2*-transfected cells were allowed to redifferentiate into neohepatocytes for 2 weeks. At the end of this period, cells were analysed for hepatic cell-specific functions (Figures 4(a)–4(c)). Neohepatocytes exhibited phase I enzyme activities. The activity of three different cytochrome P450 isoforms (1A1/2, 2D6, and 3A4) was investigated. All cells showed activity of the 3 enzymes. For all enzymes, the activity was significantly higher in neohepatocytes obtained after *SOX2* transfection than in control standard neohepatocytes. Both neohepatocytes and control cells secreted urea, and the addition of ammonium chloride (NH_4_Cl) significantly increased urea formation in all preparations. However, urea secretion was higher in neohepatocytes obtained from *SOX2-*overexpressing RMCMO (Figure 4(d)). As well, all neohepatocytes including the controls were shown to secrete glucose. To measure the ability of the cells to perform gluconeogenesis, the sodium pyruvate-containing incubation buffer was supplemented with lactate. Stimulation with pyruvate/lactate induced higher glucose secretion compared to that from cultures with pyruvate only (Figure 4(e)). As for urea formation, the gluconeogenesis was significantly higher in neohepatocytes obtained from *SOX2*-transfected RMCMO. These data show that *SOX2* transfection impacts the functional state of the redifferentiated “end product,” the neohepatocyte, most likely because of a RMCMO of higher plasticity.

### 3.5. *SOX2* Enhances the Differentiated Phenotype of Insulin-Producing Cells

**Figure 5:**
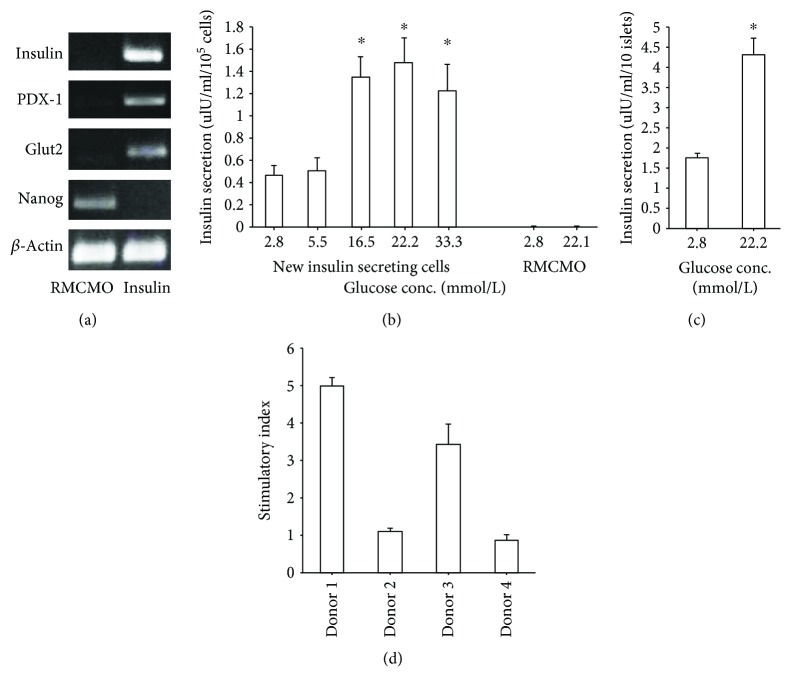
RMCMO generated from *SOX2*-transfected PBMCs are efficiently redifferentiated into insulin-producing cells. (a) Standard RMCMO or RMCMO generated from *SOX2* transfection of PBMCs (insulin) were cultured in islet cell-conditioning medium for 2 weeks and subsequently subjected to analysis of *β* cell-specific gene expression. (b) Insulin-producing cells obtained after redifferentiation of *SOX2*-transfected RMCMO (new insulin-secreting cells) or standard RMCMO were cultured with increasing glucose concentrations to determine their ability to secrete insulin. Insulin secretion was quantified from 1 × 10^5^ cells. (c) Glucose-stimulated insulin secretion in intact islets for comparison. Insulin secretion was quantified from 10 islets. (d) Insulin secretion response of insulin-producing cells obtained after redifferentiation of *SOX2*-transfected RMCMO from four different donors to glucose stimulation. The stimulatory index was calculated as glucose-stimulated secretion/basal secretion. Data are presented as mean ± SEM of *N* = 3. Statistical analysis between basal and stimulated secretions was performed by unpaired Student's *t*-test.

The *SOX2*-transfected RMCMO were also allowed to redifferentiate to insulin-producing cells. The islet cell-like differentiation was confirmed by the expression of the *β* cell-specific genes *INS*, *PDX1*, and *GLUT2*, and downregulation of *NANOG* as tested by standard endpoint PCR (Figure 5(a)). Moreover, *SOX2*-transfected cells exhibited concentration-dependent glucose-stimulated insulin secretion (Figure 5(b)), albeit the secretion level was low as compared to that of intact islets (Figure 5(c)). In addition, insulin-producing cells derived from *SOX2*-expressing RMCMO from individual donors responded quite differently to glucose stimulation (Figure 5(d)). The insulin response (stimulatory index) was calculated by dividing the secretion level at 22.2 mmol/l glucose by the basal value at 2.8 mmol/l. Some donor cells did not respond or showed only a weak response, while others displayed a high stimulatory index (Figure 5(d)).

## 4. Discussion

PBMCs were shown to be able to acquire stem cell-like features, and the resulting RMCMO have some advantageous features over other types of stem cells. These include ease of isolation from peripheral blood, absence of risk of oncogenicity because RMCMO lack telomerase expression [13], and their autologous transplantation without the need for immunosuppressive drugs. Yet, they also share with embryonic stem cells the expression of some major pluripotency genes such as *OCT3/4*, *NANOG* [12], *c-MYC* [14], and *KLF4*, the latter of which exhibits high constitutive expression in monocytes [13, 15]. However, the use of PBMCs as a cellular source for regenerative purposes in the clinical setting also has some disadvantages, i.e., a comparatively low number in peripheral blood and a limited proliferation potential in vitro [7]. To be considered as a cellular source for transplantation, it is therefore mandatory to increase cell yields by expanding them in vitro. Thus, it was the main goal of the present work to enhance the proliferation potential of PBMCs/RMCMO during culture and—at the same time—improve their redifferentiation potential towards hepatocyte-like and insulin-producing cells. We, therefore, initially attempted to induce pluripotency in plastic-adherent PBMCs by iPS technology, a technique that has opened up possibilities for diverse clinical applications. The iPS cells are able to self-renew and differentiate similarly to embryonic stem cells. They are generated by reprogramming of any kind of somatic cell of any person and therefore represent an ultimate source of personal cells. The iPS cells were initially produced using retroviral vectors encoding four transcription factors that are found at high levels in embryonic stem cells, OCT3/4, NANOG, SOX2, and KLF4. However, retroviral vectors leave a footprint in the resulting iPS cells, which could cause oncogene activation. Since this is a serious drawback for clinical applications [16], virus-free methods—like plasmid vector-mediated transfer—were developed to reduce the oncogenic risk [10]. In our hands, however, PBMCs did not tolerate the transfection procedure with the pEP4 E02S EN2K plasmid or the same vector carrying only three of the four genes. This could be attributed to either the large size of both plasmids containing the complete coding sequences of the four and three pluripotency genes, respectively, or alternatively, to the presence of a sequence that is poisonous to PBMCs. Fortunately, however, PBMCs were easily transfectable with another (smaller) plasmid containing only the *SOX2* cDNA.

Pluripotency is associated with self-renewal/proliferation. Moreover, *SOX2* has been shown to prevent exiting of the cell cycle [17, 18] which is in line with the enhanced proliferative activity in SOX2-transfected RMCMO. The proliferative activity of RMCMO derived from *SOX2*-transfected PBMCs was verified by an increase in cell counts and by nuclear Ki67 staining. The present data also showed upregulation at the mRNA level of cell cycle regulatory genes *ANAPC2*, *ABL-1*, *CDC2*, *CDK4*, and *CDK6* in RMCMO overexpressing *SOX2*. ANAPC2 controls the regulation of the G1/S and G2/M transitions, ABL-1 regulates the S-phase and DNA replication, CDC2 takes part in M-phase regulation, and CDK4 and CDK6 participate in the G1/S transition [19]. Inhibition of ANAPC2 and CDC2 has been reported to induce cell cycle arrest at G2/M [20]. Activation of the cell cycle and mitosis led to induction of proliferation as assessed by Ki67 immunofluorescence and the increases in total cell counts.

Ideally, modifications to the procedure of RMCMO generation from PBMCs should enhance not only proliferation but also the multipotent state/plasticity of these cells in a way that the resulting neohepatocytes become more hepatocyte like. Interestingly, ectopic *SOX2* expression further enhanced both *NANOG* and *OCT3/4* mRNA expression over that seen in standard RMCMO [13]. Given the already high constitutive expression of *KLF4* in monocytes [15], it is likely that the elevated protein expression of all four iPS factors in the *SOX2*-transfectants accounted for an increase in multipotency/plasticity. This, in turn, may have caused the neohepatocytes derived from *SOX2*-transfectants to more closely resemble primary hepatocytes with respect to CYP activity, urea production, and glucose metabolism.

## 5. Conclusions

Although PBMCs were refractory to transfection with an iPS plasmid, they can successfully be transfected with a plasmid carrying one pluripotency gene (*SOX2*). Ectopic *SOX2*-expression resulted in the enhancement of both RMCMO proliferation and redifferentiation into hepatocyte-like and insulin-producing cells.

## Data Availability

The data used to support the findings of this study are included within the article. Previously reported data were used to support this study and are available at PMID: 15940611, PMID: 15880050, doi:10.1111/j.1432-2277.2009.00943.x, doi:10.1186/1478-811X-10-23, doi:10.1371/journal.pone.0118097, and doi:10.1089/scd.2009.0351. These prior studies are cited at relevant places within the text as [3–7, 12].
